# Role of Multimodality Imaging in Transcatheter Structural Interventions

**DOI:** 10.14797/mdcvj.1209

**Published:** 2023-05-16

**Authors:** Amr Telmesani, Su Min Chang, Nadeen Faza, Stephen H. Little, Dipan J. Shah

**Affiliations:** 1 College of Medicine, Umm Al-Qura University, Makkah, Saudi Arabia; 2 Houston Methodist DeBakey Heart & Vascular Center, Houston, Texas, US

**Keywords:** multimodality imaging, echocardiogram, computed tomography, magnetic resonance imaging, positron emission tomography, structural intervention, transcatheter aortic valve implantation and transcatheter mitral replacement

## Abstract

Cardiac imaging is the backbone for safe and optimal transcatheter structural interventions. Transthoracic echocardiogram is the initial modality to assess valvular disorders, while transesophageal echocardiogram is best to delineate the mechanism of valvular regurgitation, preprocedural assessment for transcatheter edge-to-edge repair, and for intraprocedural guidance. Cardiac computed tomography is the modality of choice for assessing calcifications, maneuvering multiplaner reconstruction of different cardiac structures, preprocedural planning for various transcatheter valve replacement, and assessing for hypoattenuated leaflet thickening and reduced leaflet motion. Cardiac magnetic resonance imaging is best known for most accurate volumetric assessment of valvular regurgitation and chamber size quantification. Cardiac positron emission tomography is the only modality that could assess active infection through using fluorine 18 fluorodeoxyglucose radiotracer.

## Introduction

Multimodality imaging (MMI) plays a vital role in transcatheter structural interventions (TSI). Over the past 20 years, significant advancements in MMI have allowed for better procedural planning, intraprocedural guidance, and complication assessment of TSI. This review will shed light on each cardiac imaging modality and its role in TSI.

## Echocardiography

### Transthoracic Echocardiography

Transthoracic echocardiography (TTE) is the first step in the assessment of structural heart disorders, encompassing a wide variety of diseases including native and prosthetic valve stenosis and regurgitation and atrial and ventricular septal defects. When done right, TTE has tremendous potential in providing good volumetric quantification of valvular regurgitation and pulmonic-to-systemic blood volume shunt size (Qp:Qs). In addition, TTE is the modality of choice for assessing hemodynamics across suspected aortic and/or pulmonic valve stenosis.

#### Pre- And Postprocedural Imaging

Prior to undertaking any transcatheter regurgitant valvular intervention, accurate severity assessment is a must. For instance, accurate mitral regurgitation (MR) severity assessment could be difficult, especially in the presence of an eccentric/wall-hugging jet. In such situations, it is important to use the volumetric method (left ventricular stroke volume – left ventricular outflow tract stroke volume), which is best performed by injecting a left ventricular (LV) opacification agent to obtain the most accurate LV volumes. Furthermore, a good-quality LV outflow tract (LVOT) two-dimensional (2D) image to obtain an accurate LVOT diameter, and a good-quality LVOT velocity time integral, to calculate LVOT stroke volume, are needed. The volumetric method is more accurate for, assessing MR severity than proximal isovelocity surface area (PISA).^1^,^2^ For aortic or pulmonic regurgitation (transvalvular or paravalvular regurgitation) or Qp:Qs calculation, TTE study should have a good-quality 2D LVOT and right ventricular outflow tract (RVOT) views to obtain accurate LVOT and RVOT stroke volumes. Subtracting one from the other yields the regurgitation volume, while dividing one by the other yields the Qp:Qs ratio. Tricuspid regurgitation (TR) could be adequately assessed via TTE as the tricuspid valve (TV) is an anterior structure. The presence of dense triangular jet or sine wave on continuous-wave Doppler across the TV together with systolic flow reversal of the hepatic vein flow are criteria for severe TR. Quantitative assessment of TR could be done through the PISA method but its inherent flaws make it less accurate. Tricuspid valve leaflets could be sufficiently visualized via 3D TTE in patients with good acoustic windows, which is a small number of patients. Otherwise, this assessment is heavily relied on short-axis transgastric views of the transesophageal echocardiogram.^3^

#### Intraprocedural

In contemporary transcatheter aortic valve implantation (TAVI), transesophageal echocardiography is not mandatory. TTE is used immediately after valve implantation in the procedure room to confirm normal transvalvular gradient and velocity, assess for paravalvular leak, and to rule out complications such as pericardial effusion.

### Transesophageal Echocardiography

Transesophageal echocardiography (TEE) has high spatial and temporal resolutions (0.6 cm and 15 msec, respectively). Therefore, it is the best modality to assess for mechanisms of native and prosthetic valvular regurgitations, except for the mechanical aortic valve, as shadows would prevent an adequate assessment. In this scenario, cardiac computed tomography with angiography (CTA) would best serve the purpose.

#### Pre- And Postprocedural Imaging

TEE is usually the next modality in assessing MR severity if TTE is equivocal. This is carried out by evaluating the spectral Doppler systolic reversal of the pulmonary vein, calculating the 3D vena contracta (severe if ≥ 0.4 cm^2^),^4^ and visualizing the MR jet or paravalvular regurgitation in mechanical mitral valves (MVs), as significant shadows prohibit adequate assessment via TTE. Another useful role of TEE is assessment for endocarditis and its complications, such as aortic root abscess. While prosthetic valve endocarditis is not rare, endocarditis of transcatheter valves is uncommon. For TAVI, it ranges from 0.4% to 2.8% depending on the time of implantation: for instance, early (< 3 months post-procedure) carries the highest incidence, while late (> 1 year after) is the lowest.^5^

**Figure 1:**
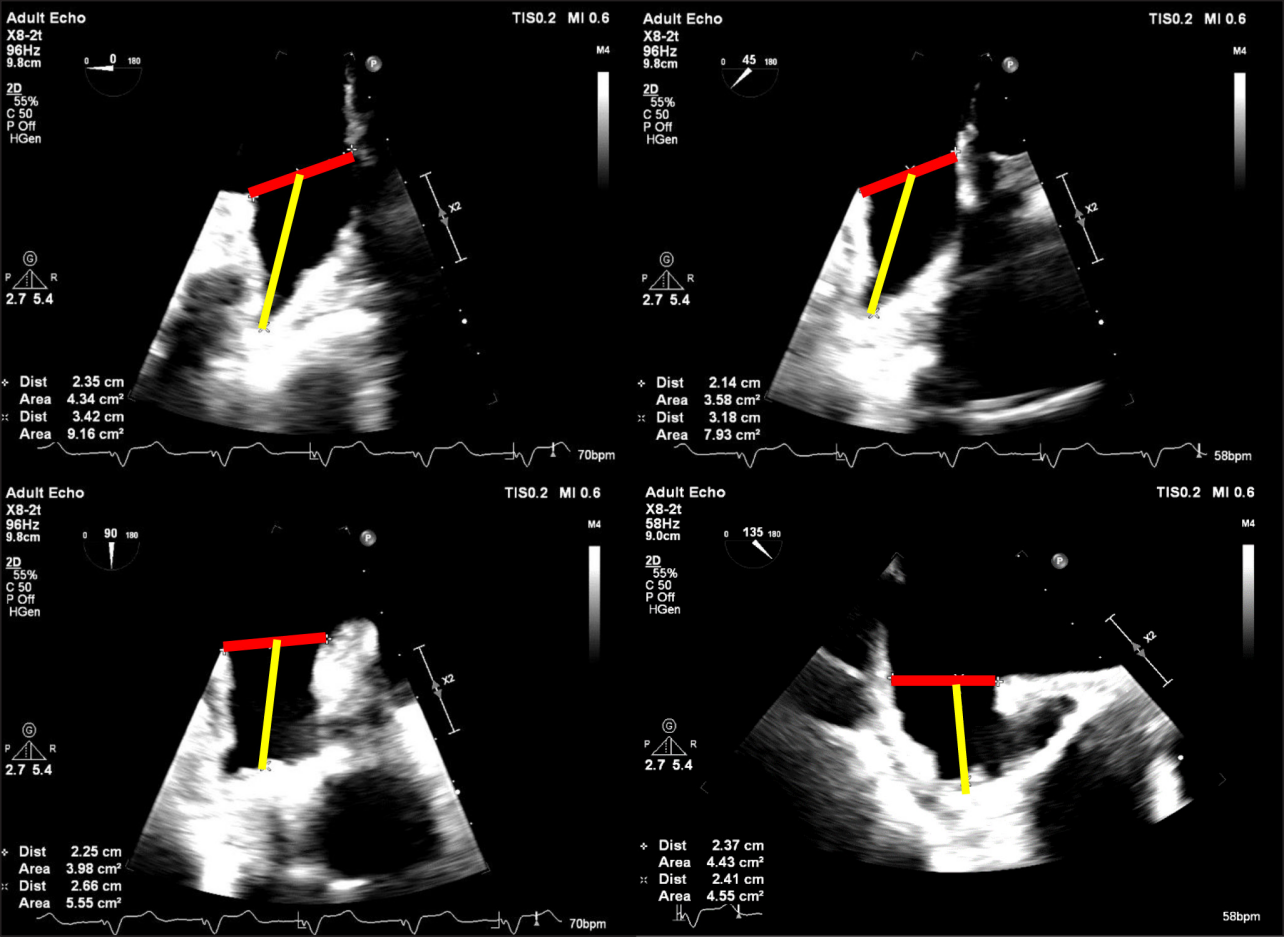
Transesophageal echocardiogram demonstrating left atrial appendage measurements on different planes (0, 45, 90 and 135 degrees). The horizontal red line represents the width and the yellow line represents depth.

**Figure 2:**
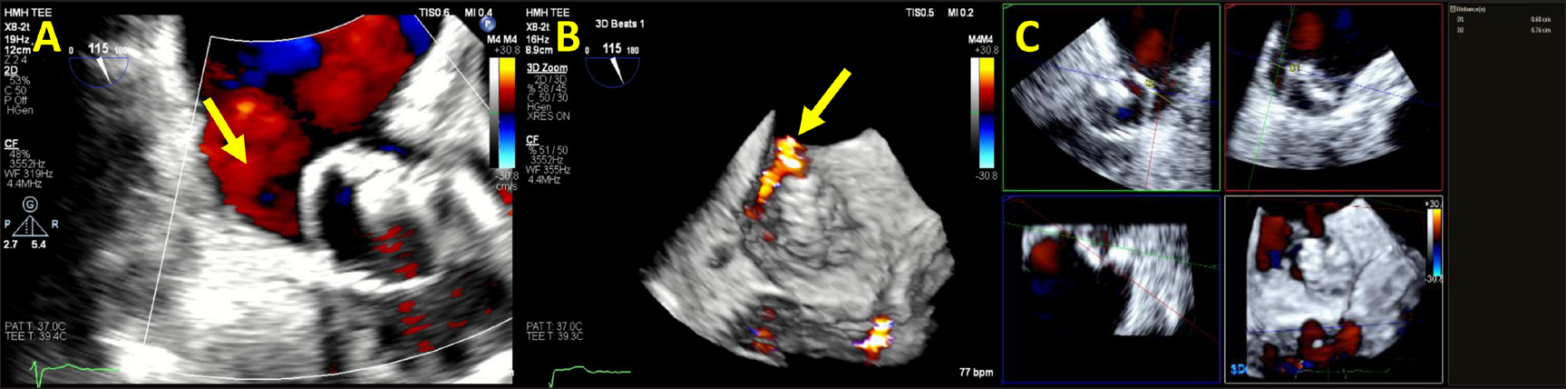
Transesophageal echocardiogram showing **(A)** Video clip of 2D color Doppler peri-Watchman device leak (yellow arrow). **(B)** Video clip of 3D color Doppler transesophageal echocardiogram showing peri-Watchman device leak (yellow arrow). **(C)** 2D multi-planer reconstruction of the Watchman device demonstrating the size of the peri-device leak at 0.8 × 0.6 cm. See Figure 2 A videoclip at https://youtu.be/LlqtscEZVR4 and Figure 2 B videoclip at https://youtu.be/F0qbwF45LLQ.

Utilizing TEE for preprocedural evaluation is key in determining the feasibility of MV edge-to-edge repair (TEER). Evaluation of MV TEER involves the following: (1) posterior leaflet length should be ≥ 7 mm; (2) diastolic MV area measured on a 2D multiplanar reconstruction (MPR) of the 3D MV acquisition should be ≥ 4 cm^2^; (3) assessing location of flail/prolapsed segment (medial segments, A3/P3, are less favorable); (4) assessing fossa ovalis height (favorable to have at least 4 cm from the fossa to the plane of the MV, especially in Barlow’s MV); and (5) evaluating baseline transvalvular mean gradient (MG). In mitral and aortic paravalvular regurgitation closure procedures, TEE confirms the presence and location of the leak. On the right side of the heart, tricuspid valve transcatheter interventions, such as transcatheter valve replacement, TEER and annuloplasty are being used more frequently in recent years. TEE is used for preprocedural assessment and is a cornerstone for TEER feasibility.^3^ Beside valvular assessment, TEE is important in preprocedural planning for left atrial appendage occlude devices (LAAOD). Multiple views are obtained at 0, 45, 90, and 135 degrees to assess the appendage width and depth and to rule out appendage thrombus (Figure 1). Post-LAAOD implantation, TEE is used to evaluate for peridevice leak via 2D color Doppler and 3D MPR (Figure 2). A peridevice leak, including small ones (≤ 5 mm in size), is associated with higher risk of stroke.^6^ Thus, it should be adequately assessed.

#### Intraprocedural Imaging

**Figure 3:**
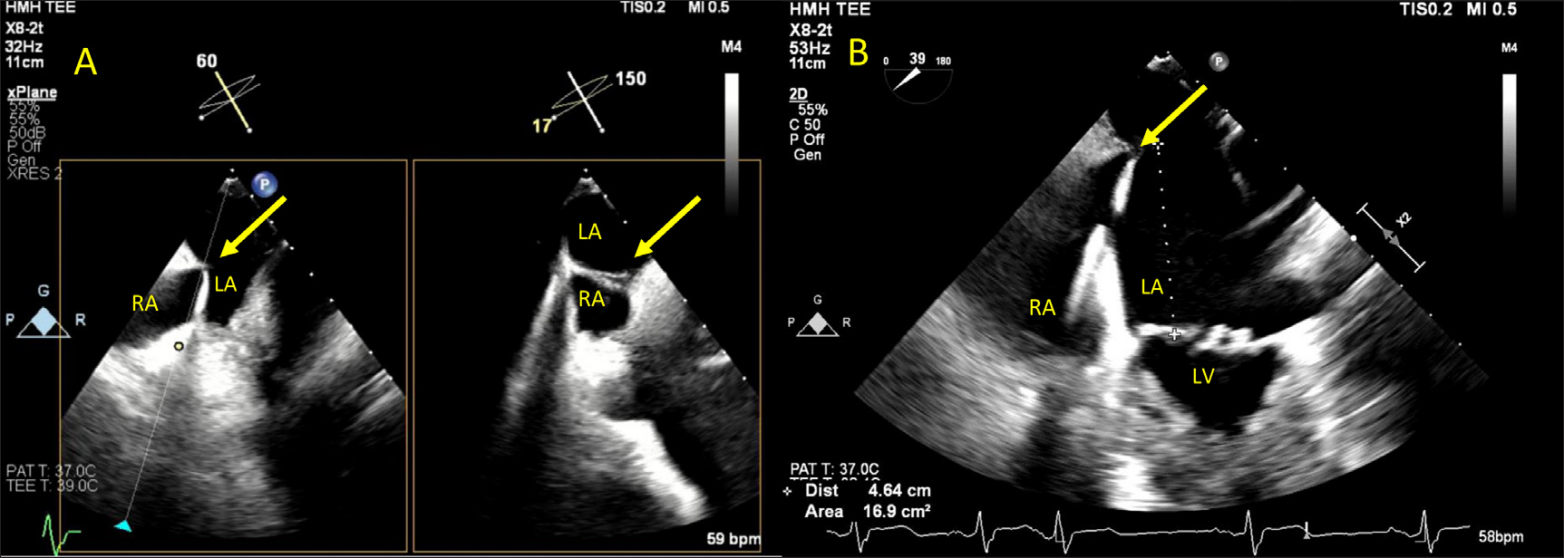
**(A)** Versacross sheath with Baylis radiofrequency wire to cross the interatrial septum (yellow arrow). **(B)** Measuring the distance between the crossing wire and the mitral valve plane. Optimal distance ≥ 4 cm. See Figure 3 A videoclip at https://youtu.be/1b_bV_QfKd8.

**Figure 4:**
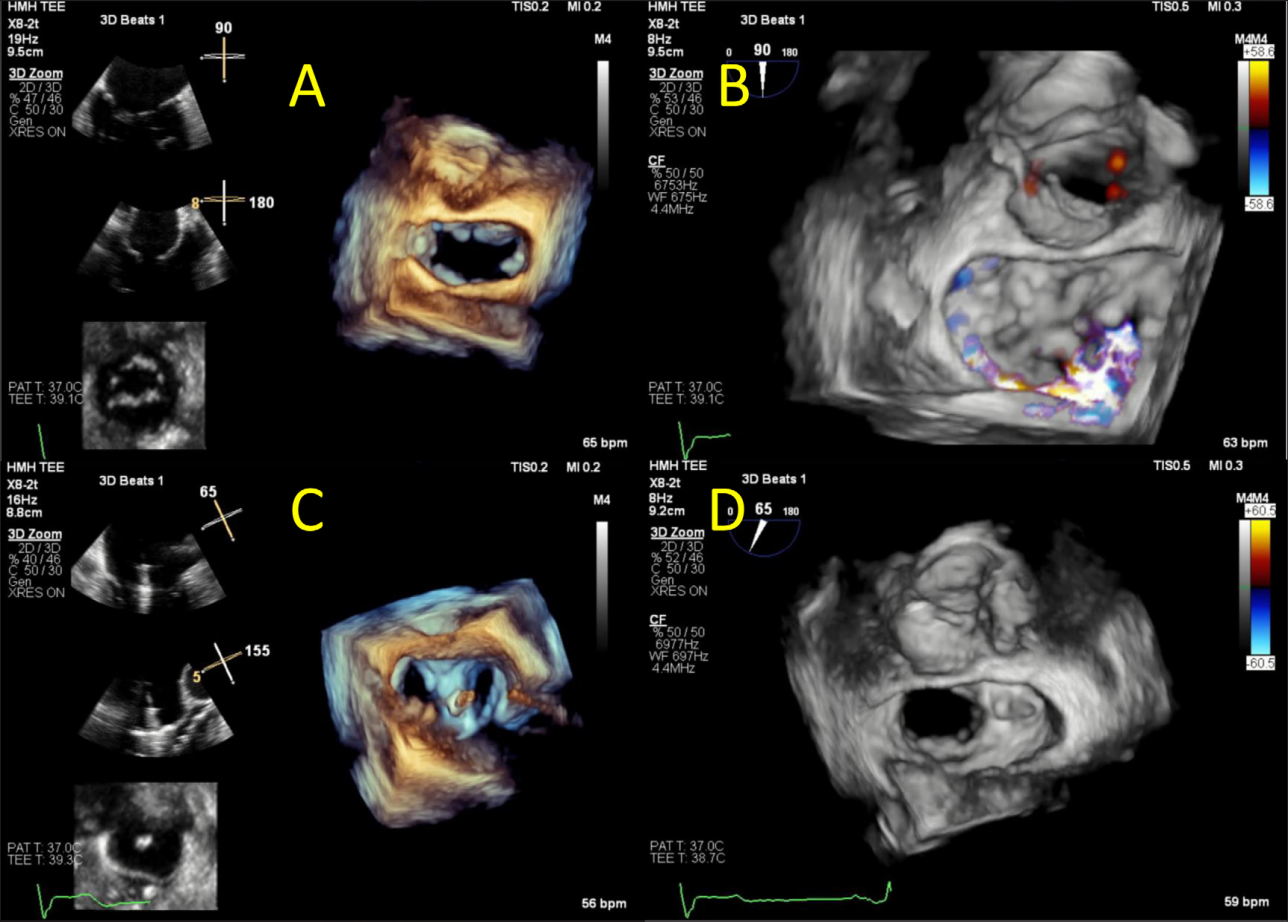
Transesophageal echocardiogram showing **(A)** 2D and 3D video clip of the mitral valve showing medial aspect of A2 and A3 prolapse. **(B)** 3D color Doppler video clip of the mitral valve showing posteriorly directed mitral regurgitation. **(C)** 2D and 3D video clip of the mitral valve showing the guide catheter and MitraClip position after grasping A2 and P2 scallops. **(D)** 3D color Doppler video clip of the mitral valve after deploying the MitraClip. Mild residual MR noted. See Figure 4 A videoclip at https://youtu.be/NRvFQL6UBJA, Figure 4 B videoclip at https://youtu.be/wTsxav-JLbo, Figure 4 C videoclip at https://youtu.be/xH8RG0NrHp4 and Fig4D videoclip at https://youtu.be/yMQjrnIhM_0.

**Figure 5:**
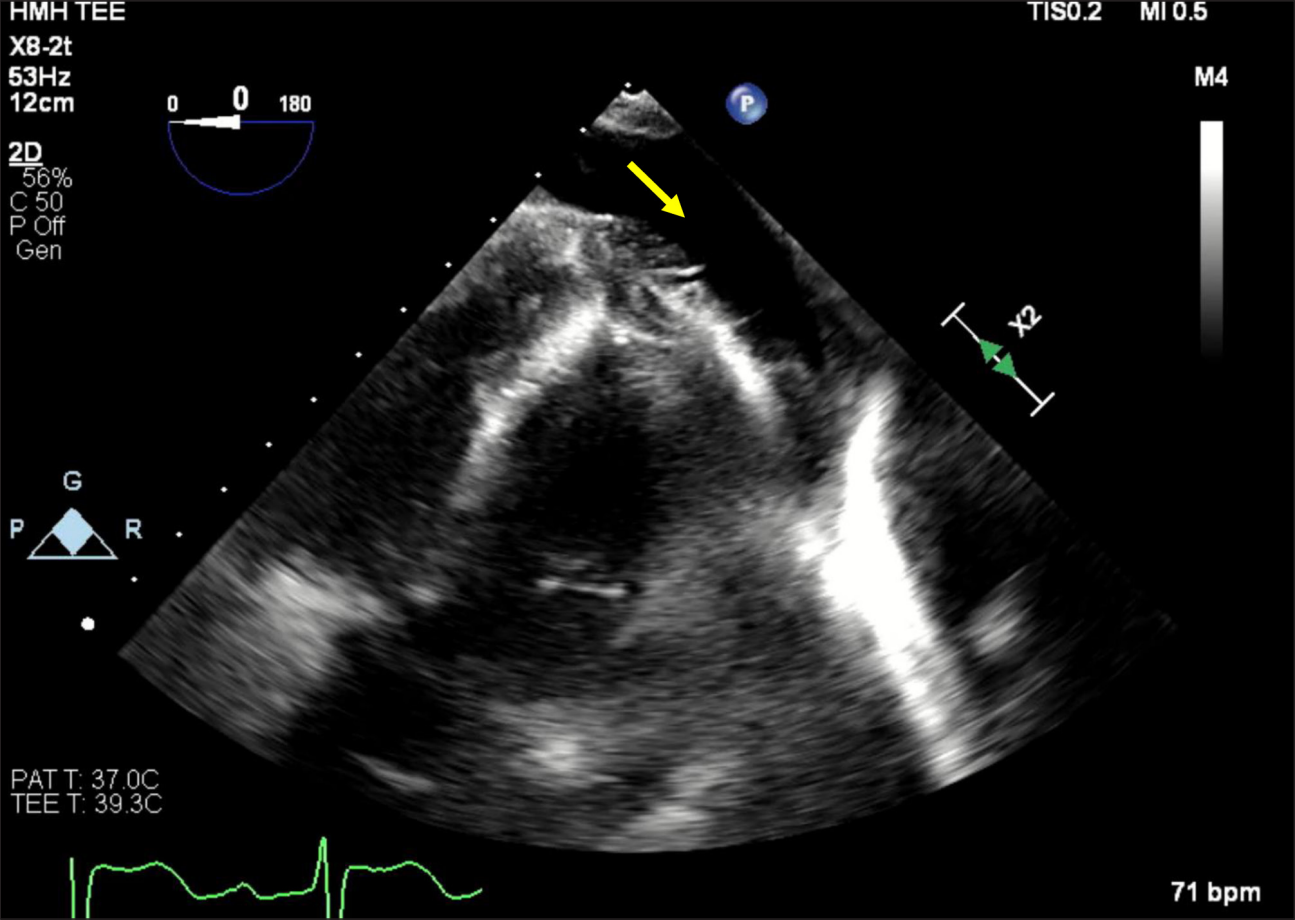
Transesophageal echocardiogram, transgastric view showing moderate pericardial effusion (yellow arrow) during MitraClip procedure that required urgent pericardiocentesis. See Figure 5 videoclip at https://youtu.be/_4LF1h95oR0.

**Figure 6:**
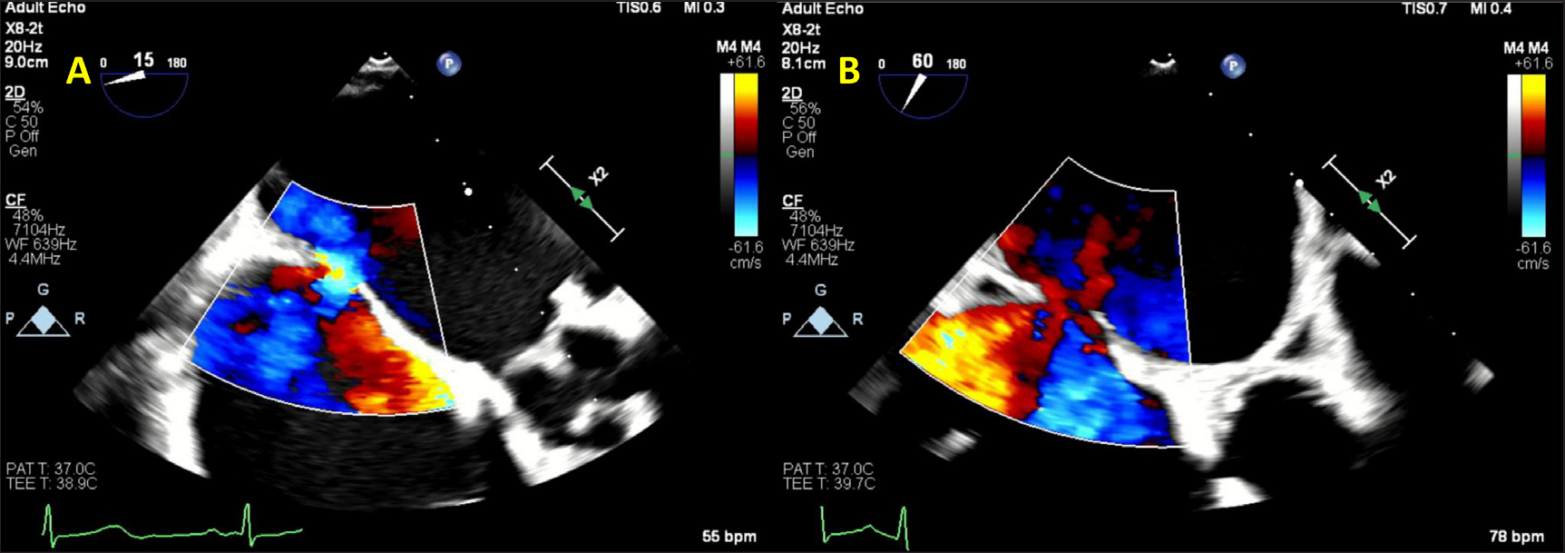
Transesophageal echocardiogram showing **(A)** residual iatrogenic atrial septal defect (ASD) with bidirectional shunt from prior transcatheter mitral valve replacement that was complicated by paravalvular leak (PVL). **(B)** During attempt to close the PVL, the patient developed intractable hypoxia and was found to have worsening ASD with bidirectional shunt due to enlargement of the ASD size. See Figure 6 A videoclip at https://youtu.be/Zfbb80XK-jI and Figure 6 B videoclip at https://youtu.be/em48KZ8hbJY.

**Figure 7:**
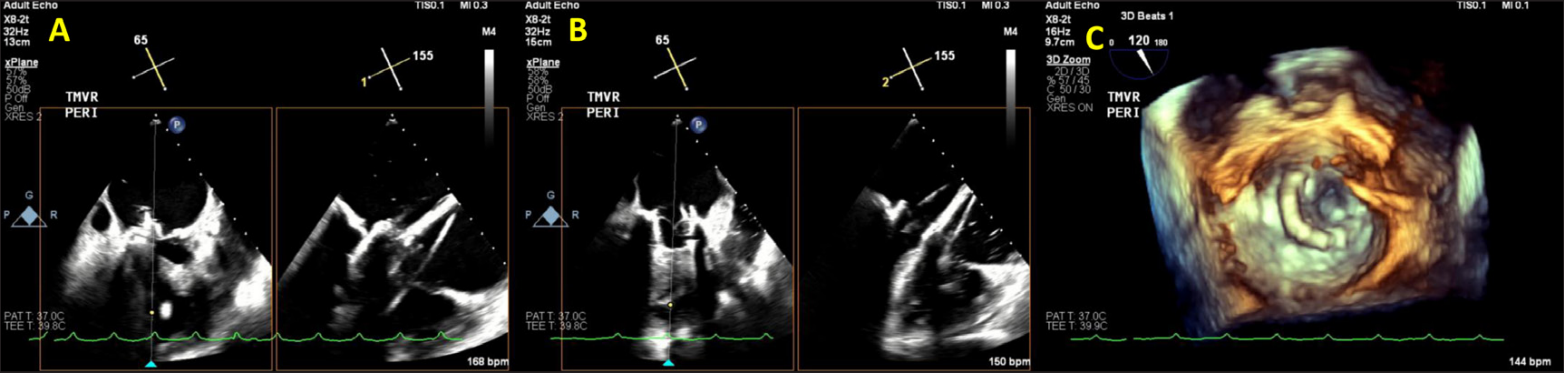
Transesophageal echocardiogram showing **(A)** transcatheter mitral valve implantation (TMVR) implantation. **(B)** Immediately after TMVR implantation, normal leaflet motion is noted. **(C)** 3D reconstruction demonstrating normal prosthesis leaflet motion. See Figure 7 A videoclip at https://youtu.be/ksmMxnu-oSk, Figure 7 B videoclip at https://youtu.be/r8dTvavy0jY and Figure 7 C videoclip at https://youtu.be/Yp4q9dn_os8.

TEE is the modality of choice for intraprocedural guidance given its inherent portability, real-time imaging, and good temporal and spatial resolutions. For MV TEER guidance, the interatrial septum is crossed through the fossa ovalis on the superior and posterior aspects (Figure 3). Then, the guide catheter is positioned in the area of interest (Figure 4). After initial deployment of the clip, TEE is used to assess for residual MR and transvalvular MG. If the clip position is satisfactory, the clip is released and a second interrogation is performed to assess for MR, which could worsen due to malpositioning during clip release. Once the guide catheter is removed, the last step is to assess for complications such as pericardial effusion (zero-degree, four-chamber view, and transgastric view) (Figure 5) and enlargement of the iatrogenic atrial septal defect (ASD), which could lead to significant bidirectional shunt (Figure 6). TEE is also used to guide transcatheter mitral valve replacement (TMVR) (Figure 7). If the neo-LVOT is anticipated to be small on the planning CTA for TMVR, a LAMPOON procedure (laceration of the anterior mitral leaflet to prevent LVOT obstruction), which is done via TEE guidance, is performed to allow for blood flow through the valve struts into the LVOT. For TAVI, intraprocedural TEE is not mandatory. However, in some cases where coronary obstruction risk is high, especially in valve-in-valve cases, the BASILICA (Bioprosthetic or Native Aortic Scallop Intentional Laceration to Prevent Iatrogenic Coronary Artery Obstruction) procedure could be done and is optionally facilitated via TEE.^7^ Also, assessing for new wall motion abnormality is another important sign for coronary obstruction. Regarding TV, TEE is important in guiding various transcatheter procedures such as TEER, annuloplasty, and transcatheter tricuspid valve replacement. LAAOD implantation also uses TEE for guidance intraprocedurally; however, some centers use intracardiac echocardiogram, or both. Upon implantation, the device should increase the osteal diameter of the appendage by 8% to 20%.^8^

## Computed Tomography

Over the past 20 years, the cardiac computed tomography (CT) field has expanded exponentially. Currently, it is a key modality for planning preimplantation of transcatheter devices. One of the great features of CT is its ability to produce isotropic images (ie, a true 3D image), where all pixels’ dimensions are equal. This allows for easy MPR manipulation of any structure given that CT has good spatial resolution (a typical high-resolution cardiac CT is 0.6 × 0.6 × 0.6 cm). Although the temporal resolution is lower than TEE and cardiac magnetic resonance imaging (around 66-75 msec for dual-source CT machines), it allows for adequate assessment of leaflet motion, cardiac chamber volume, and ejection fraction if retrospective gating is performed. In fact, CT could replace fluoroscopy in the assessment of mechanical valve leaflets motion.

### Pre- and Postprocedural Imaging

**Figure 8:**
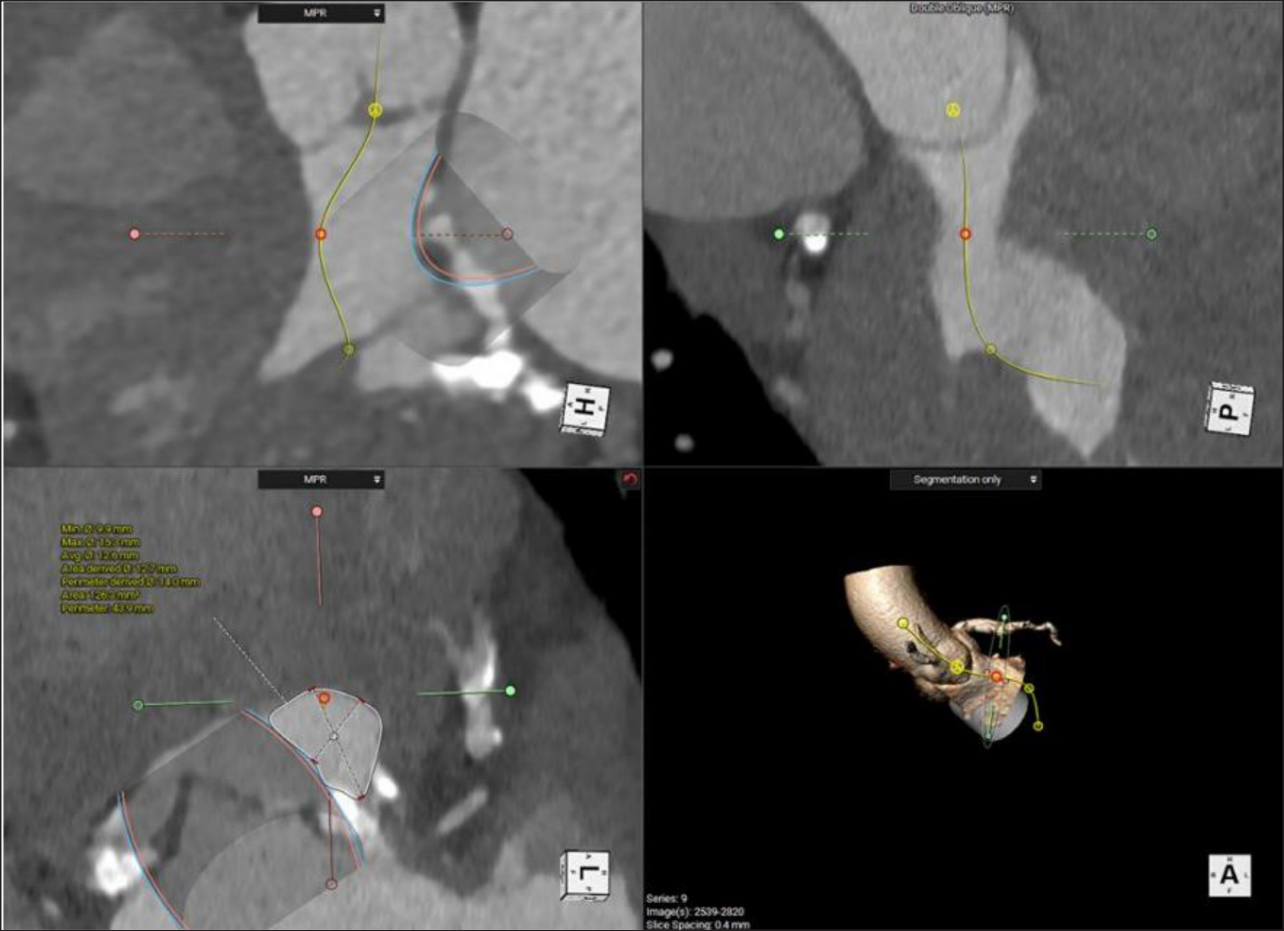
Cardiac computed tomography showing multiple views of the neo-left ventricular outflow tract (neo-LVOT) after implanting the virtual mitral valve. It measured 1.3 cm^2^, which is high risk for LVOT obstruction.

**Figure 9:**
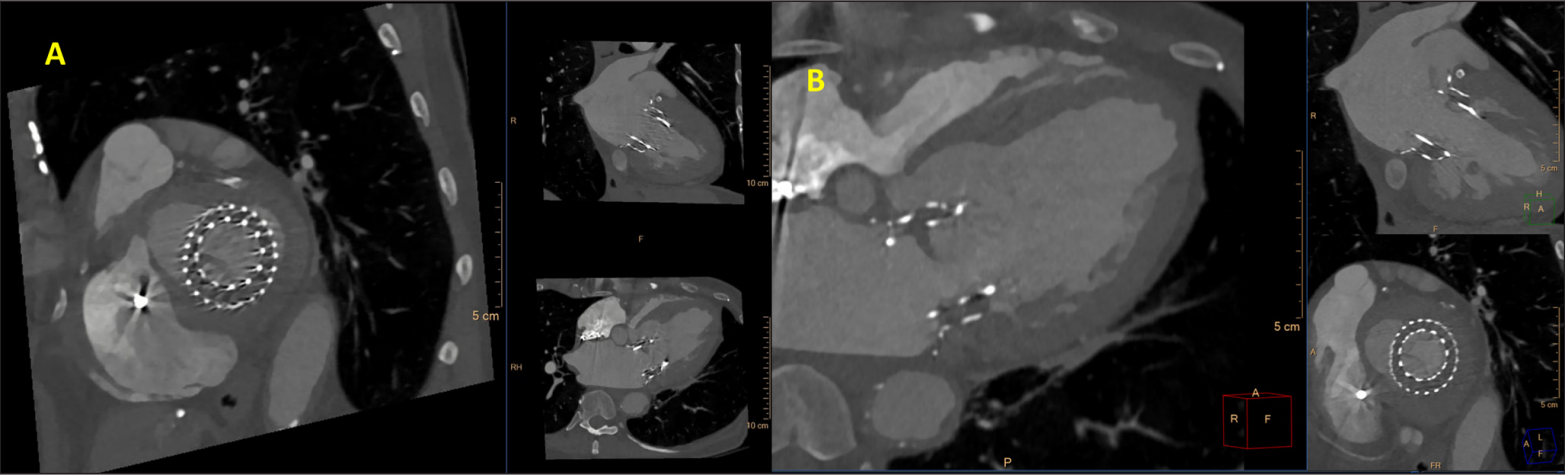
Cardiac computed tomography showing **(A)** video clip of the transcatheter mitral valve replacement (TMVR) with restricted leaflet motion. **(B)** Hypoattinuated leaflet thickening (HALT) >75% of the TMVR leaflet. See Figure 9 A videoclip at https://youtu.be/1O5I4xX9rGk.

**Figure 10:**
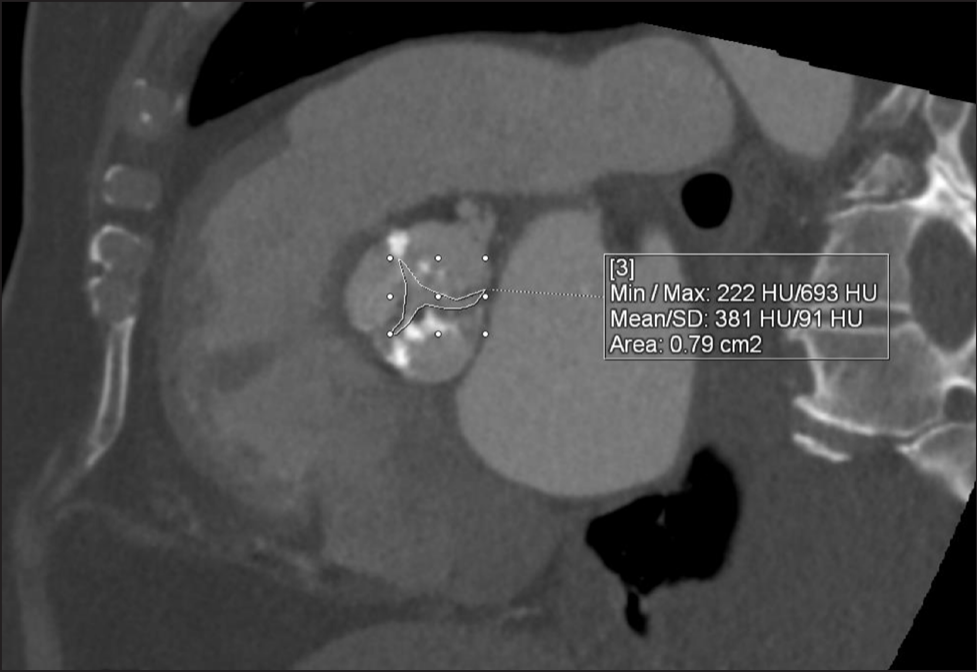
Cardiac computed tomography showing severe calcific aortic valve stenosis (valve area 0.79 cm^2^) via planimetry.

**Figure 11:**
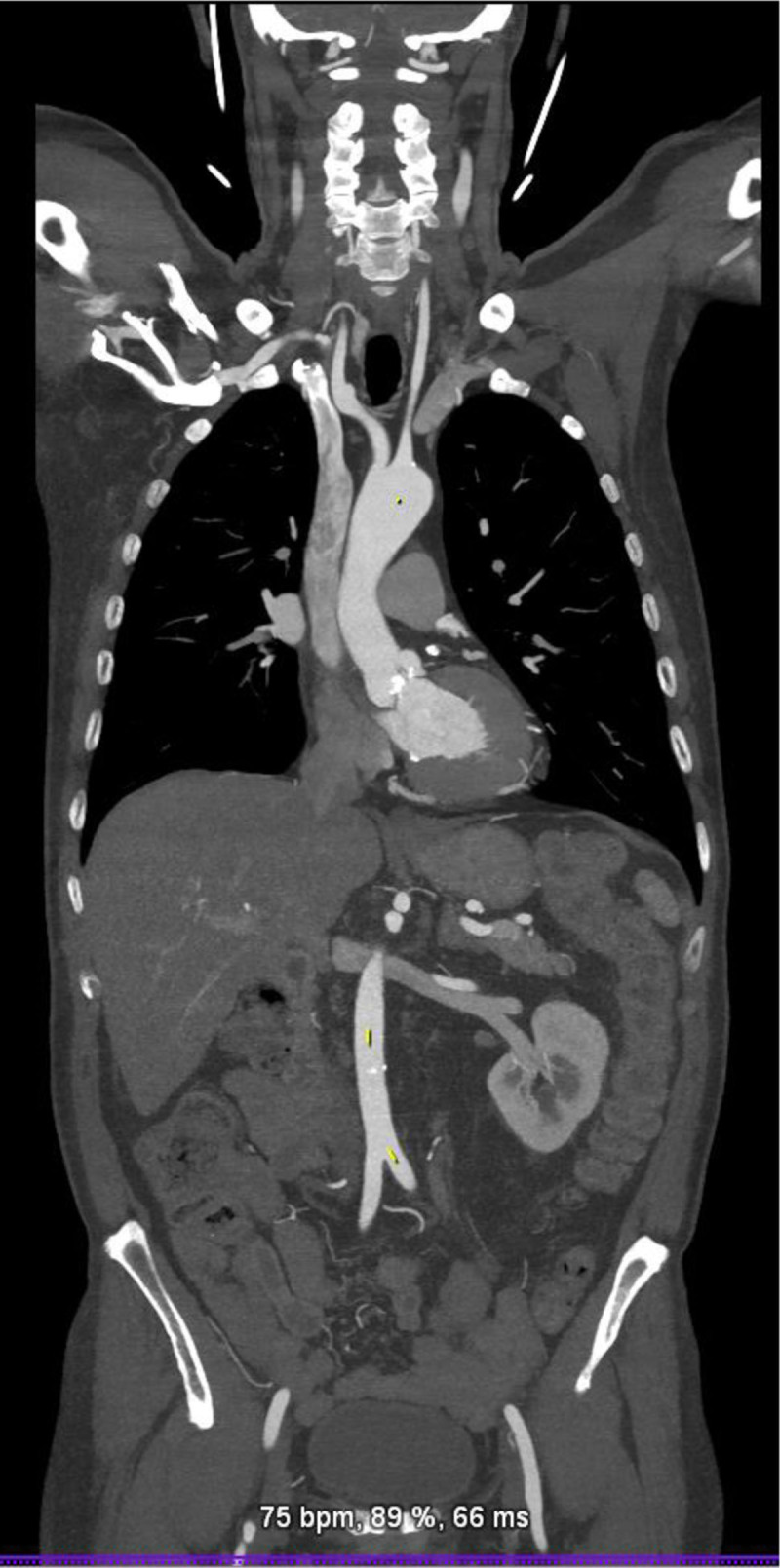
Cardiac computed tomography showing the vascular access from the level of the carotid arteries to superficial femoral arteries.

**Figure 12:**
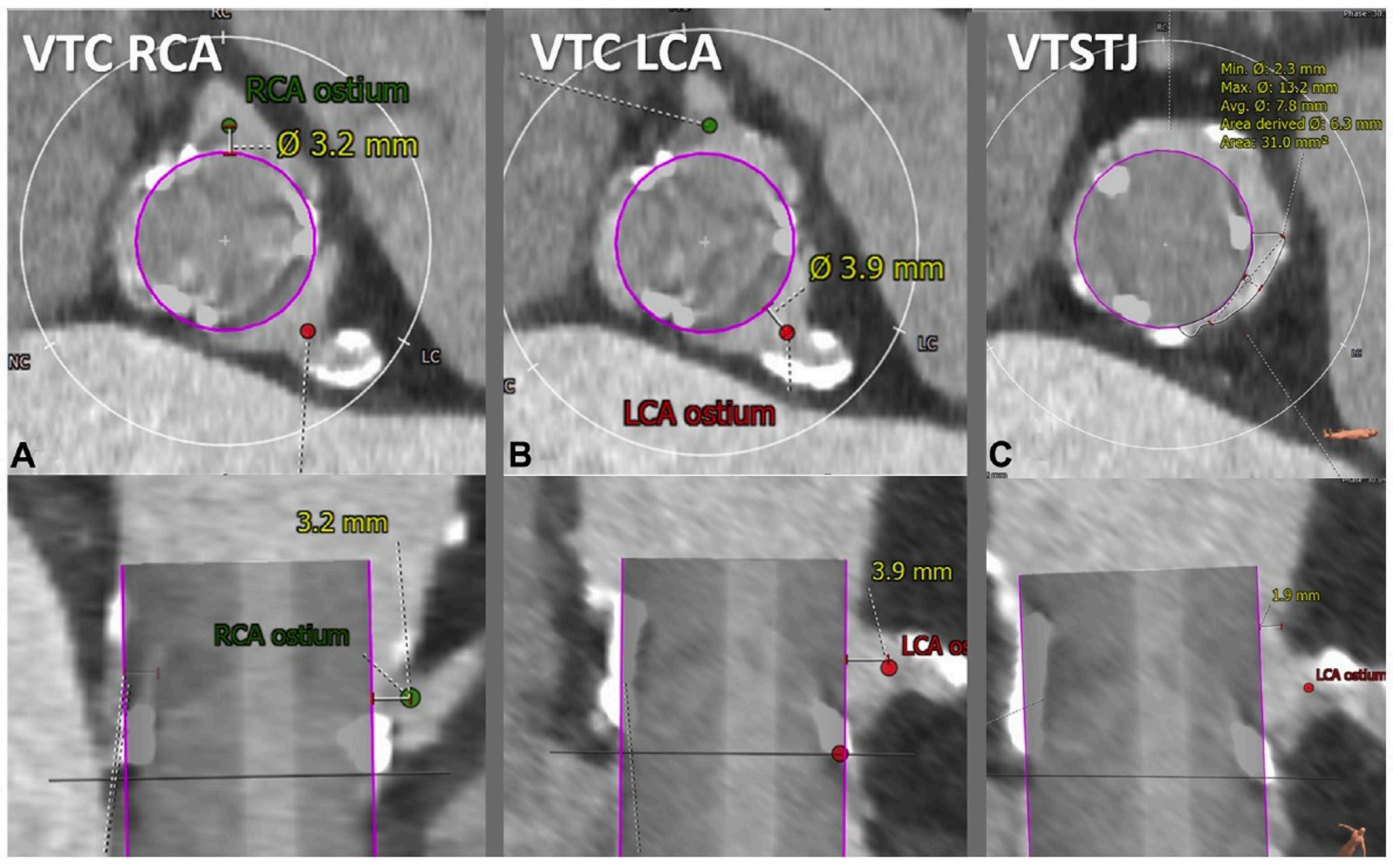
Cardiac computed tomography showing the virtual valve–to–coronary (VTC) distance, which is measured in 2 orthogonal planes (top, axial; bottom, longitudinal). Representative VTC distance measurement for **(A)** a right coronary artery (RCA) and **(B)** left coronary artery (LCA). The valve-to-sinotubular junction (VTSTJ) distance is measured in orthogonal planes **(C)**: axial (upper) and longitudinal (lower), when the sinotubular junction is lower than the height of the transcatheter aortic valve replacement device. Reprinted with permission.^9^

In TMVR cases, CT is used to assess the diastolic MV area, mitral annulus area, degree of calcification, to rule out LAA thrombus, and most importantly to estimate the area of the neo-LVOT by inserting a virtual valve and tracing the remaining area of the LVOT. If neo-LVOT is ≤ 1.6 cm^2^, it is considered a high risk for significant LVOT obstruction (Figure 8). This has important implications for procedural planning since the operator needs to perform a LAMPOON procedure to relieve the obstruction. Postprocedural TMVR assessment for hypoattenuated leaflet thickening (HALT) and reduction in leaflet motion (RELM) could be performed with superior image quality (Figure 9). Classical CT preprocedural planning is for TAVI. Computed tomography allows for an accurate anatomical systolic aortic valve area via planimetry (Figure 10), assessment of the degree of calcification (visually and via calcium score), and vascular access assessment from the carotids all the way to the superficial femoral arteries (Figure 11). Furthermore, CT is instrumental in assessing for coronary obstruction risk. Coronary height, which is the distance from the aortic annulus to the coronary ostia, should generally be > 10 mm to 12 mm to mitigate coronary obstruction risk. Other factors, especially in case of valve-in-valve implantation, are the virtual valve-to-coronary distance (VTC) and virtual valve-to-sinotubular junction distance (VTSTJ) (Figure 12).^9^ A VTC < 3 mm and/or VTSTJ < 2 mm (the averaged value) increases risk for coronary obstruction. These numbers would help in assessing the feasibility of the BASILICA procedure.

**Figure 13:**
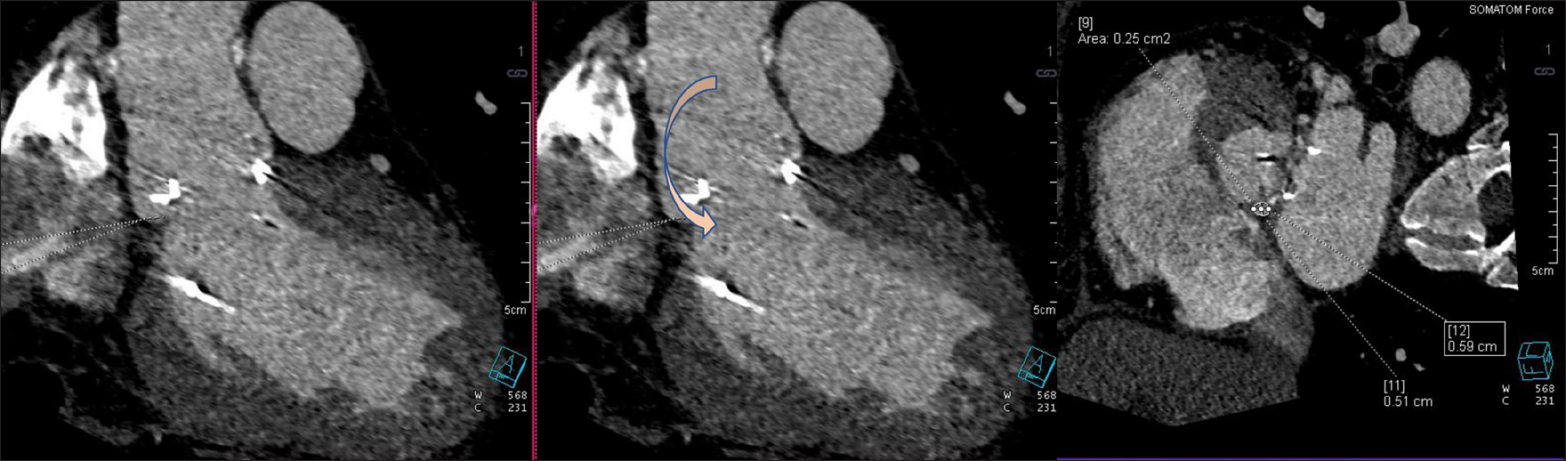
Cardiac computed tomography showing surgical aortic valve with para-valvular leak (arrow).

**Figure 14:**
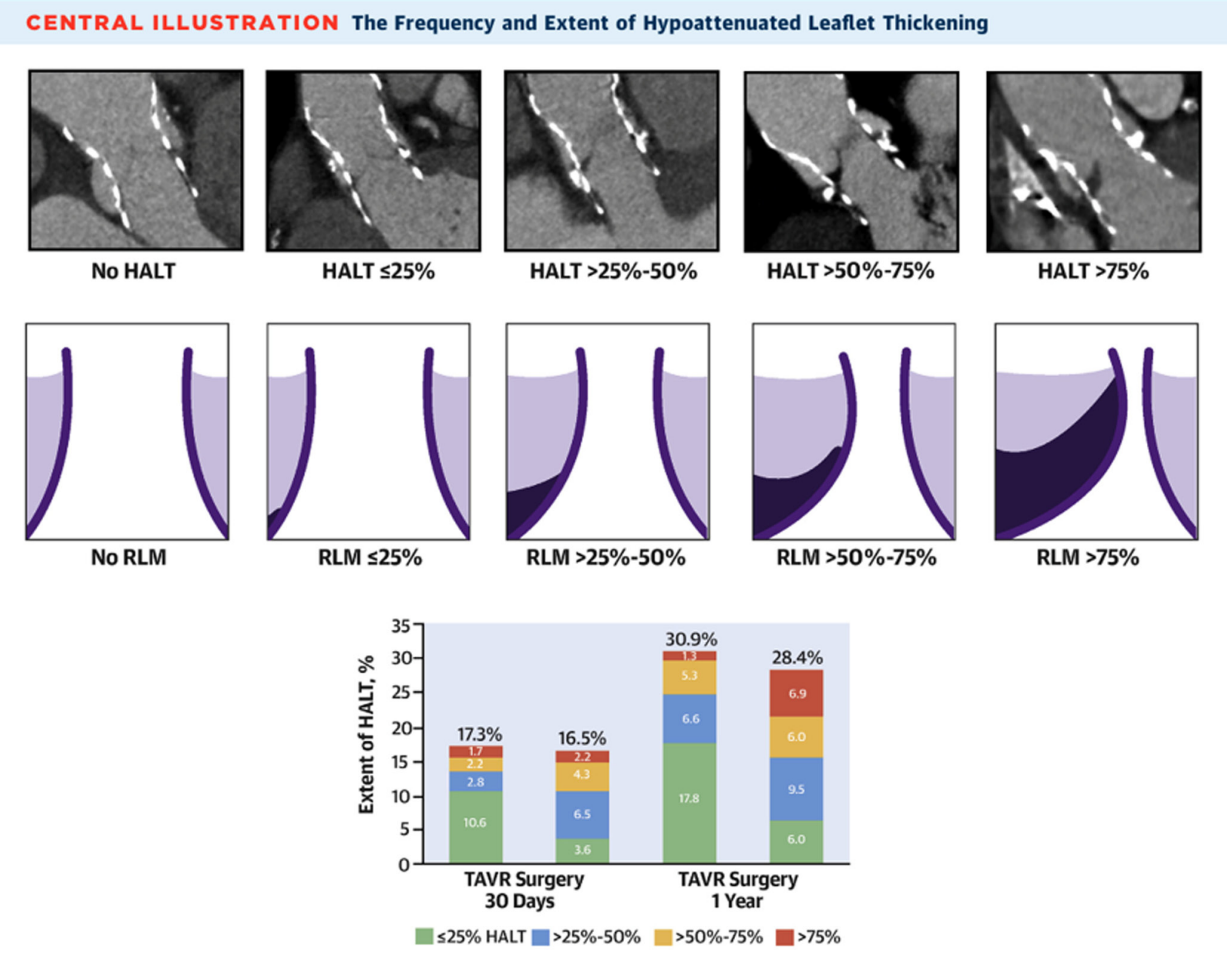
Cardiac computed tomography (CT) showing grading of hypoattenuated leaflet thickening (HALT). Reprinted with permission.^13^

**Figure 15:**
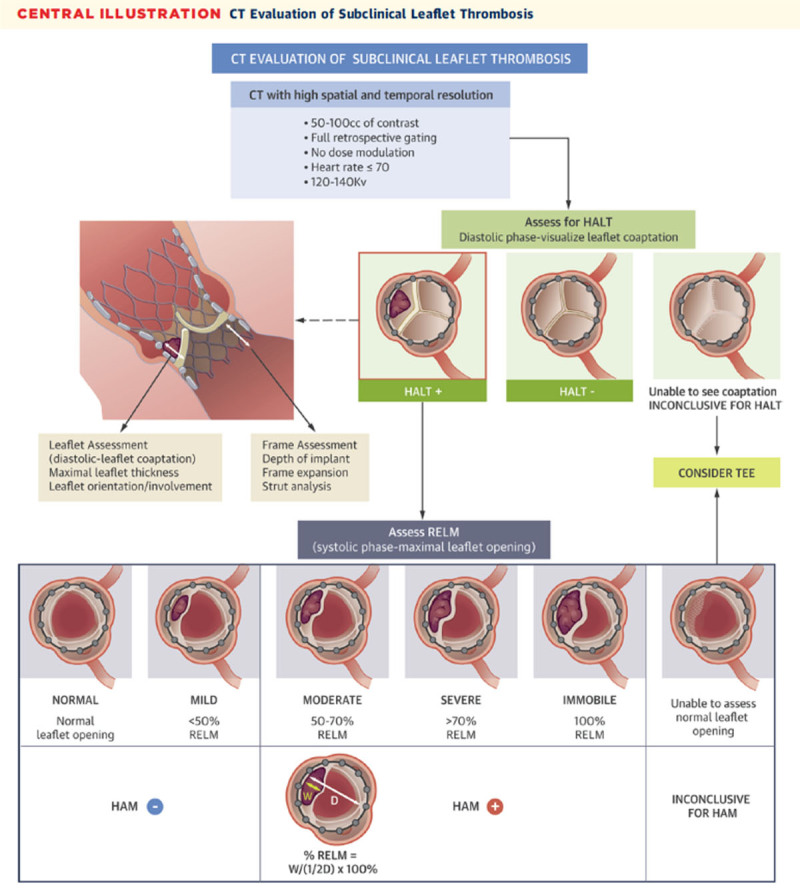
Illustration showing hypoattenuated affected motion (HAM) and calculating the percentage of reduced leaflet motion (RELM). Reprinted with permission.^14^

Postprocedural TAVI paravalvular regurgitation, if noted on echocardiography, is assessed for location and size via CT (Figure 13). HALT is a CT finding that could suggest subclinical thrombus formation of the bioprosthetic leaflet. In TAVI, early HALT (within 30 days) is found in 10% to 20% of newly implanted valves.^10^,^11^ There is conflicting data regarding mortality and Doppler hemodynamics across the prosthesis in HALT cases, with some reports suggesting higher mortality and worse hemodynamics in 1 to 3 years after implantation.^10^,^12^,^13^ HALT is graded from 0% to > 75% depending on the extent of thickness from the base to the tip of the leaflet (Figure 14). HALT could exert RELM, and if the latter was significant (≥ 50% RELM), it is then called hypoattenuated affected motion (HAM). The RELM percentage is calculated on the short-axis view of the TAVI during systole (maximum leaflet opening) (Figure 15).^14^ Protocoling of the cardiac CT angiography for assessment of HALT and RELM/HAM should be performed with retrospective gating and postprocessing reconstruction increment of ≤ 10% phases. These parameters provide adequate cine clips to assess leaflet motion and to increase the likelihood of capturing the phase/interval of maximum leaflet opening. Cardiac CT could also be used for LAAOD preimplantation planning. Wider-range systolic imaging, in addition to a delayed acquisition to confirm the absence of LAA thrombus, is needed. LAA orifice measurements should be done on MPR during maximal LAA dilatation, which is usually during mid-to-late LV systole.^15^ The atrial side of the LAAOD should not exceed the LAA orifice plane; to achieve this, the device landing zone should be situated deeper into the LAA.^16^ Post-LAAOD implantation evaluation is conducted to assess thrombus formation on the atrial side of the device and detect any peridevice leaks. The presence of LAA contrast in the delayed acquisition is consistent with a peridevice leak. The 2D MPR allows for locating and sizing the orifice of the leak.^16^

## Cardiac MRI

Cardiac MRI (CMR) provides the most reliable volumetric quantification for regurgitant heart diseases, including native, surgical, and transcatheter valves. Contemporary CMR uses a steady-state free precession (SSFP) sequence, which offers a quick acquisition time and great signal-to-noise ratio (SNR) that translates into improved image quality. However, SSFP acquisition has a high susceptibility artifact that prevents adequate prosthesis assessment. Using gradient echo acquisition allows for reduced void metal artifacts and more reliable volumetric calculations. CMR has a better temporal resolution than CT (typical CMR cine acquisition is about 35-45 msec) but with a lower special resolution (1.5 × 1.5 × 6 mm). Therefore, CMR is not the modality of choice for visualizing the presence of endocarditis or accurately defining tricky mechanisms of regurgitation, such as small focal/commissural flail. In general, most of the recent structural surgical and transcatheter devices (such as valve replacements, valve clips, repair rings, LAAODs, interatrial septum occlude devices, and LAA clips) are compatible with MRI scans under certain scanning parameters. For each product specification, one should refer to www.mrisafety.com.

### Pre- and Postprocedural Imaging

**Figure 16:**
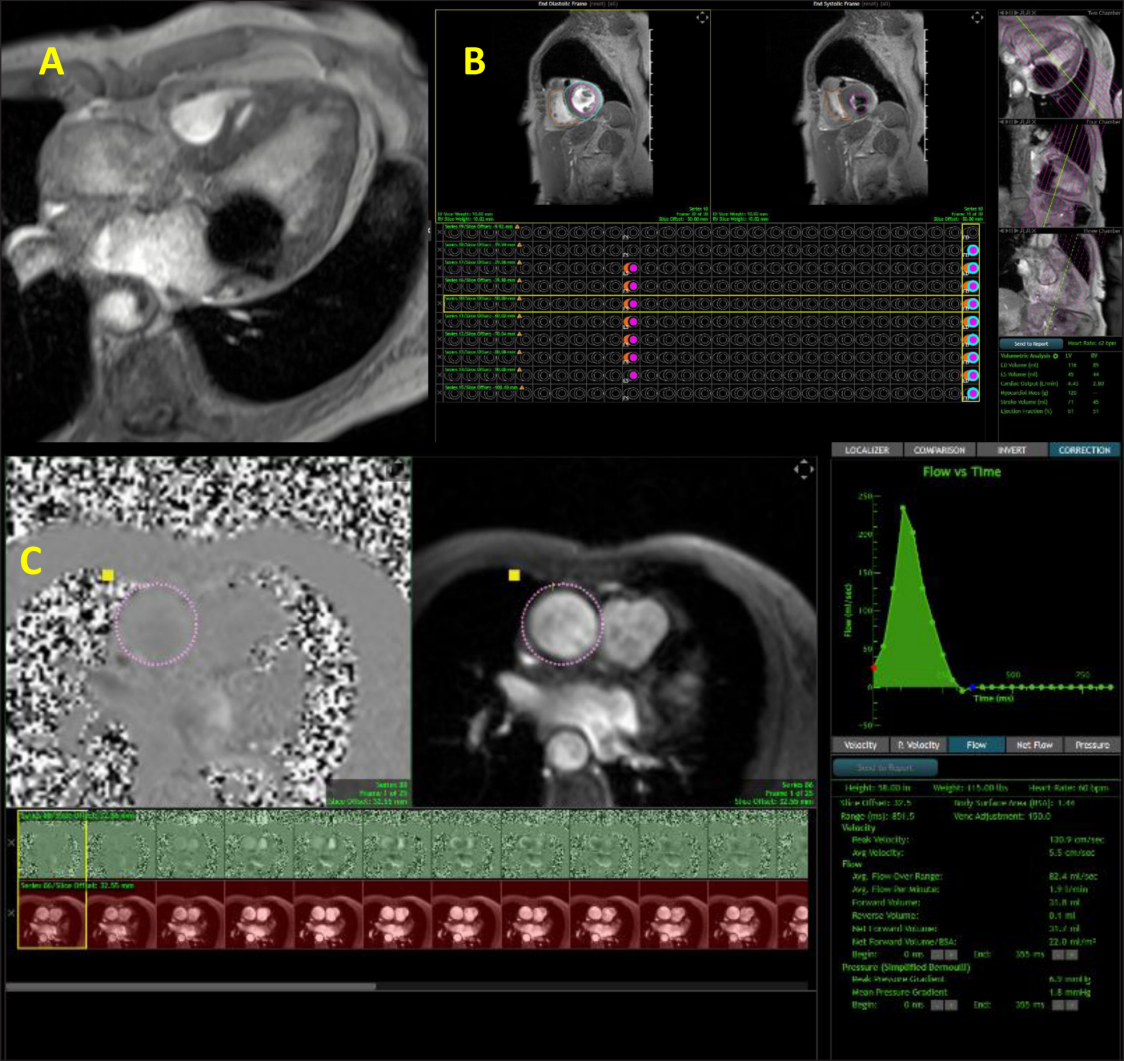
Cardiac magnetic resonance showing **(A)** Gradient echo cine clip void artifact with MitraClip noted within the mitral valve, **(B)** left and right ventricular volumes, and **(C)** velocity encoding of the aortic valve to calculate the aortic forward flow. Residual mitral regurgitation was calculated at 39 mL (moderate MR). See Figure 16 A videoclip at https://youtube.com/shorts/dzBitLYytBE.

**Figure 17:**
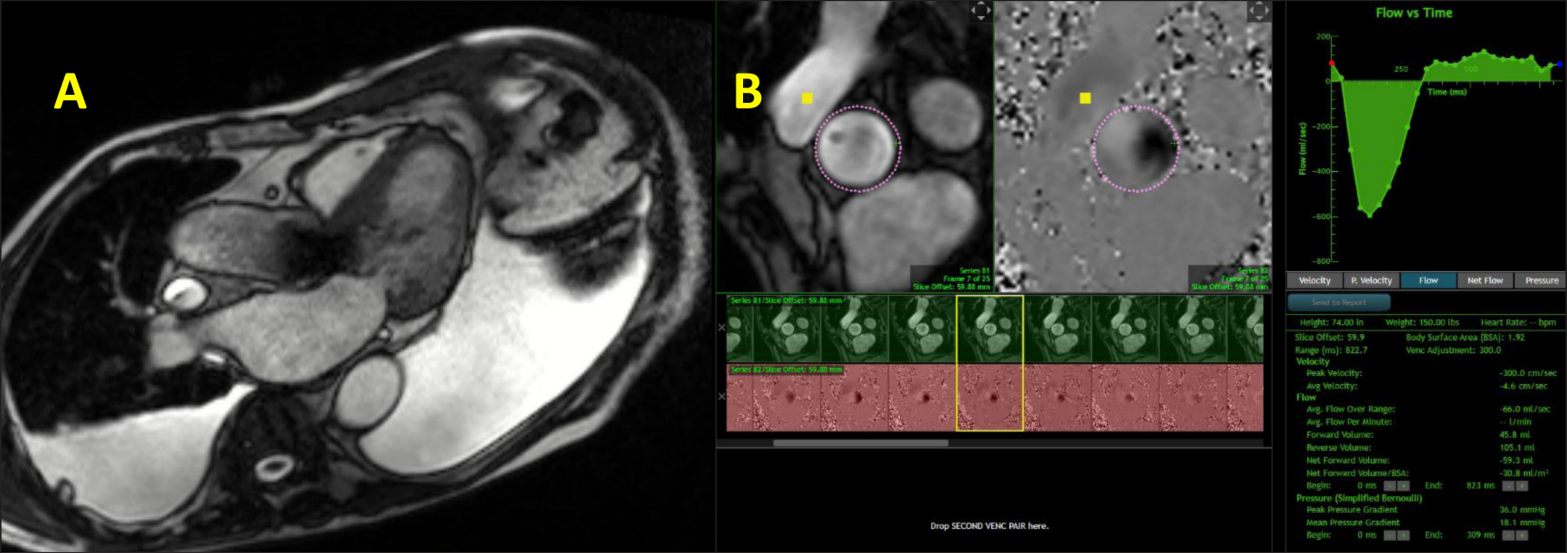
Cardiac magnetic resonance showing **(A)** gradient echo clip with transcatheter aortic valve implantation prosthesis. **(B)** Velocity encoding of the aortic valve to calculate the aortic forward flow and aortic regurgitation; the latter was estimated at 46 mL. See Figure 17 A videoclip at https://youtu.be/QzSLwsdtRxA.

**Figure 18:**
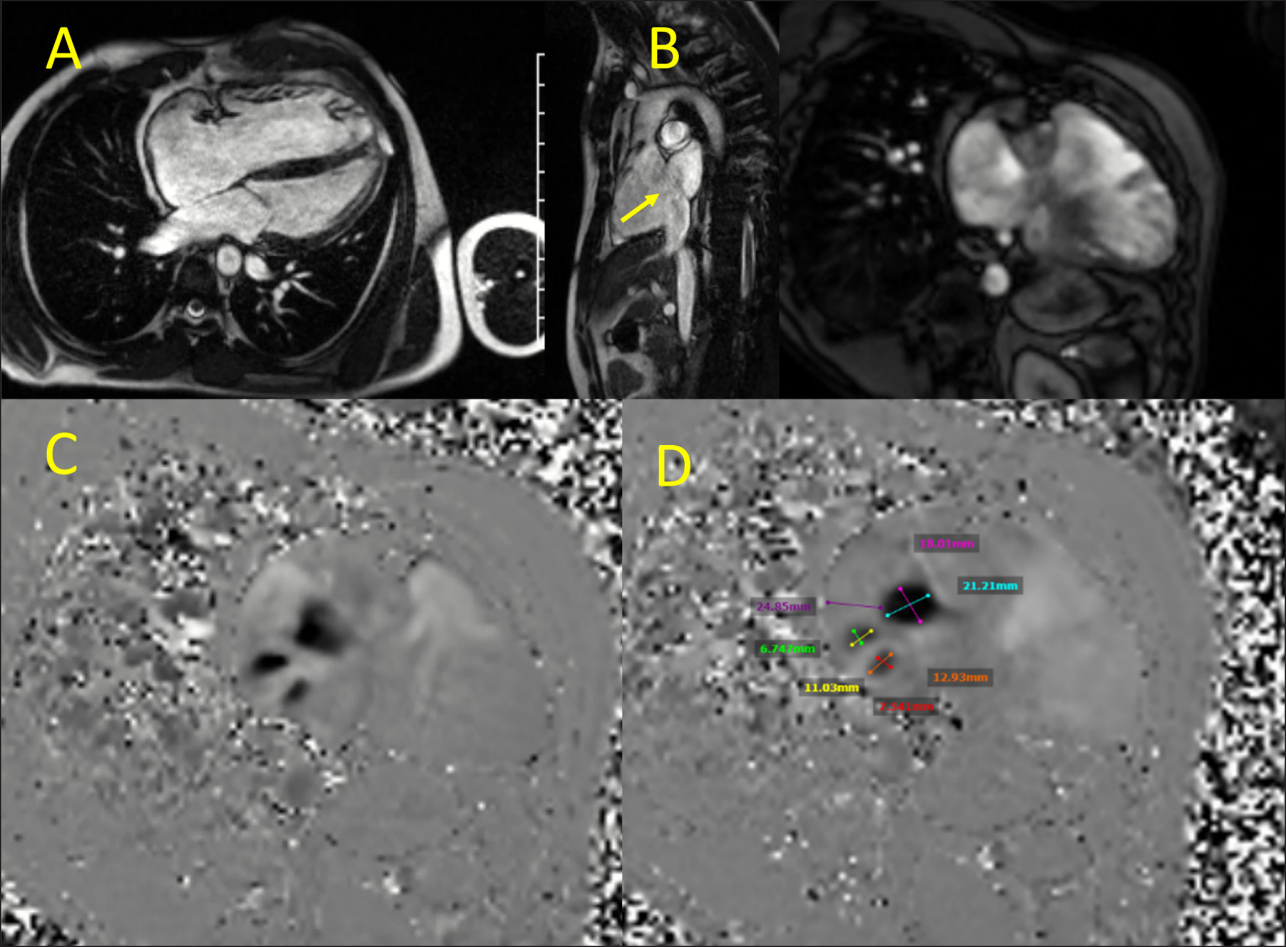
Cardiac magnetic resonance showing **(A)** steady state free precession (SSFP) cine clip showing right atrial and ventricular enlargement, **(B)** secundum atrial septal defect (yellow arrow), **(C)** SSFP cine clip 2-chamber view of the right ventricle, and **(D)** velocity encoding, 2-chamber view of the right ventricle showing 3 atrial septal defects with their measurements. See Figure 18 Top Center videoclip at https://youtu.be/8dMYzYOpQ2k, Figure 18 Top Left videoclip at https://youtu.be/zg6snVhWxMg and Figure 18 Top Right videoclip at https://youtu.be/_ZQrmcqkrdU

**Figure 19:**
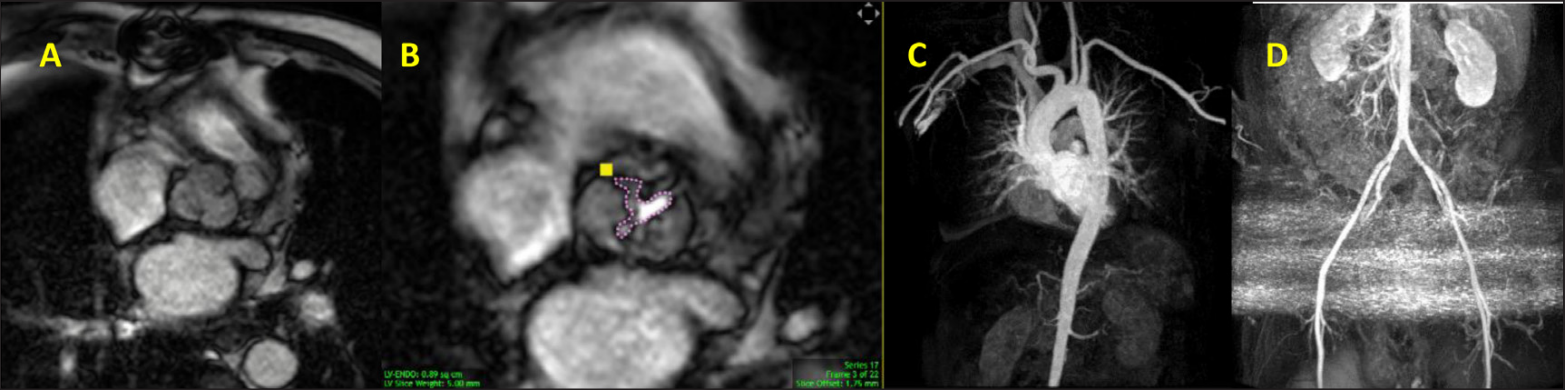
Cardiac magnetic resonance showing **(A)** gradient echo clip short axis view of the aortic valve. Stenosis is noted. **(B)** Planimetry of the aortic valve demonstrating severe aortic stenosis with aortic valve area of 0.9 cm^2^. (**C** and **D**) Maximal intensity projection of the aorta and lower extremity arteries. See Figure 19 A videoclip at https://youtube.com/shorts/sVsBIuobsUU.

Volumetric estimation for residual MR in MitraClip cases is done by calculating the aortic forward flow and subtracting it from the calculated LV stroke volume (Figure 16). TAVI paravalvular leak, on the other hand, is calculated by subtracting the pulmonic forward flow from the aortic forward flow (Figure 17). For secundum ASD, dimensions could be measured off the velocity encoding sequence (Figure 18), and Qp:Qs is measured by dividing the pulmonic forward flow by the aortic forward flow. Another useful indication for CMR is in the pre-TAVI evaluation of patients with significant renal impairment. It provides accurate valve area via planimetry that is comparable to CT, as well as vascular access evaluation (Figure 19). If scar assessment is not indicated, then intravenous ferumoxytol can be used, which is not nephrotoxic. On the other hand, if scar assessment is needed, group II gadolinium-based contrast agents can be used safely since nephrogenic systemic sclerosis is rare with the newer gadolinium agents.^17^,^18^

## Cardiac Positron Emission Tomography (PET)

**Figure 20:**
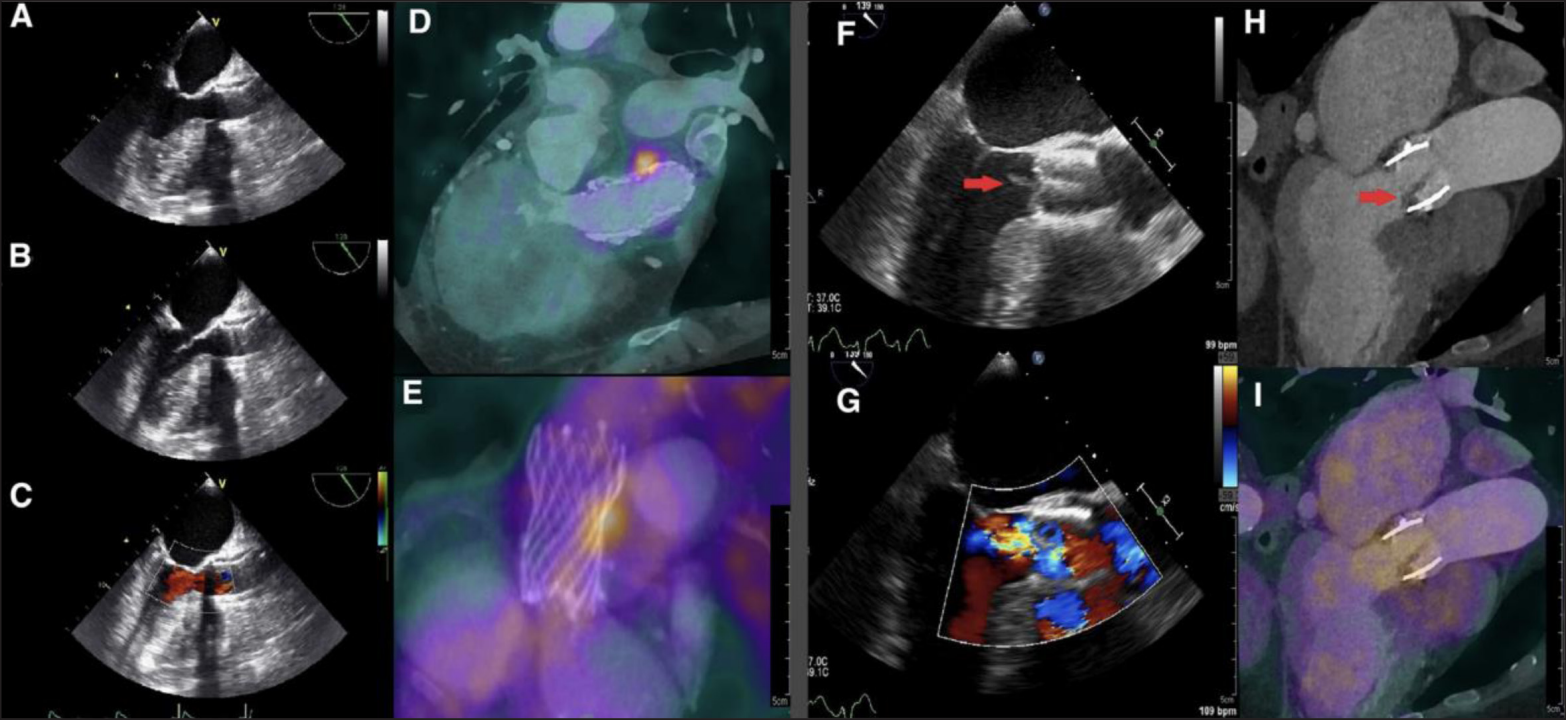
Two cases of one positive PET/CT and one negative PET/CT for TAVI-IE. Case 1 (A to E): A 75-year-old female with suspected Corevalve TAVI-IE underwent a TEE without signs of endocarditis (**A to C**). PET/CT images (**D/E**) demonstrated focal FDG uptake alongside the Corevalve as positive sign of TAVI-IE. This case was previously published as a case report. Reprinted with permission.^19^ Case 2 (F to I): An 81-year-old female with suspected Edwards-Sapien TAVI-IE underwent a TEE (**F/G**) with a vegetation on the aortic valve and mild aortic regurgitation. CTA demonstrated thickening of the aortic valve leaflets (**H**) as possible signs of vegetation. However, PET/CT images (**I**) showed no focal 18F-FDG uptake on the leaflets. This was explained by the low inflammatory activity and 2 weeks of intravenous antibiotic therapy prior to the PET/CT scan. Reprinted with permission.^20^ PET/CT: positron emission tomography/computed tomography; TAVI-IE: transcatheter aortic valve implantation-infective endocarditis; FDG: fludeoxyglucose F18; TEE: transesophageal echocardiography; CTA: computed tomography angiography

Cardiac PET has an emerging role in diagnosing endocarditis of prosthetic valves using a fluorine 18 fluorodeoxyglucose (FDG) radiotracer. Inflammatory cells uptake FDG despite fasting state and high-fat diet (Figure 20).^19^ This type of study should be performed at centers with experience and excellence as such cases, especially resolving infections or partially treating ones, are difficult to assess.

## Key Points

- Multimodality cardiac imaging is a cornerstone in transcatheter structural interventions.
- Different cardiac imaging modalities complement each other.
- It is important to know the strengths and limitations of each modality to better serve patients.
